# Changes in ultrasound and bioelectrical-derived body composition parameters in response to training load during pre-season in professional male road cyclists

**DOI:** 10.1186/s13102-025-01213-3

**Published:** 2025-07-02

**Authors:** Andrea Giorgi, Francesco Campa, Martino V. Franchi, Maurizio Vicini, Borja Martinez-Gonzalez

**Affiliations:** 1 Department of Internal Medicine, Specialist Medicine and Rehabilitation, Azienda USL Toscana-Southeast, Siena, Italy; 2 VF Group-Bardiani-CSF-Faizanè Professional Cycling Team, Bibbiano, Italy; 3 Department of Biomedical Sciences, University of Padua, Padova, Italy; 4 Kent and Medway Medical School, University of Kent, Canterbury, UK

**Keywords:** Body composition, Pre-season, Professional cyclists, Performance, Training

## Abstract

**Purpose:**

The pre-season is commonly a period during which cyclists implement interventions to optimize performance, training, and body composition. Ultrasonography and bioelectrical impedance vector analysis (BIVA) can serve as efficient methods to track changes in body fat and lean soft tissue. This study aimed to longitudinally monitor changes in subcutaneous adipose tissue thickness and fluids during the pre-season in response to training load.

**Methods:**

Twelve professional male cyclists participated in the study. Body composition and field performance data were collected on three occasions over three months during the pre-season: December (T1), January (T2), and February (T3).

**Results:**

Significant reductions in total subcutaneous adipose tissue thickness (*p* = 0.001; ES = 0.45) and fat mass percentage (*p* = 0.005; ES = 0.38) were observed. Phase angle increased (*p* < 0.001; ES = 0.68), while the impedance vector decreased (*p* < 0.001; ES = 0.66). Training metrics, including distance, elevation gain, and workload, increased. Improvements were recorded in 5-min, 20-min, and 60-min mean maximal power outputs.

**Conclusions:**

Professional road cyclists exhibited reduced adiposity, particularly in the lower limbs, alongside increases in soft tissue and extracellular fluids during the pre-season. Monitoring body composition during the pre-season using ultrasonography and BIVA can provide valuable insights for coaches, sports scientists, and medical staff, enabling them to tailor training loads and optimize cyclists’ readiness for the competitive season.

## Introduction

The professional road cycling competitive season typically begins at the end of January and concludes in mid-October, encompassing 30,000–35,000 km across 65–90 days of competition [1]. Following the season, cyclists undergo a short detraining period (off-season) lasting 3–4 weeks before entering the pre-season phase. During this phase, training resumes with the objective of preparing for the upcoming competitive season. Variations in training and dietary habits during the pre-season can lead to fluctuations in cyclists’ physical capabilities [2, 3]. As cycling is a weight-sensitive sport, riders aim to optimize their power-to-weight ratio to enhance performance [4]. In this context, regular body composition assessments are crucial for monitoring the effectiveness of training prescriptions.

Body composition is a key determinant of athletic performance, and cyclists may benefit from lower body mass and reduced body fat levels [5]. Historically, body fat estimation relied heavily on skinfold measurements. However, advancements in technology now allow for the quantification of body fat and other tissues, such as lean muscle mass, using a variety of methods ranging from sophisticated laboratory instruments to practical field-based tools. In this context, the use of B-mode (brightness mode) ultrasonography (US) has gained attention as an accurate and efficient method for measuring subcutaneous adipose tissue thickness (SAT) [6–8]. Similarly, bioelectrical impedance analysis (BIA) is increasingly recognized as a valuable tool for assessing changes in body fluids [9, 10]. These advancements are of growing interest among sports practitioners aiming to optimize athlete performance.

Ultrasonography (US) is a medical imaging technique that has gained popularity as a tool for assessing body composition. It has demonstrated potential for monitoring subtle fluctuations on SAT, particularly in athletic populations. By producing a two-dimensional image, US enables detailed body composition assessment, allowing practitioners to distinguish between different tissue types with greater precision [6–8].

Excessive SAT thickness can hinder cyclists’ performance, whereas optimal levels of soft tissue and a correct fluid balance among intra and extracellular spaces influence their performance [4, 5]. In recent decades, bioelectrical impedance vector analysis (BIVA) and bioelectric phase angle (PhA) have proven to be valuable tools for assessing soft tissue and fluid changes [11]. BIVA allows the interpretation of the raw parameters resistance (R) and reactance (Xc), plotting them as a vector within a graph [11]. PhA is calculated as an arctangent of the ratio between R and Xc and was found to accurately reflect the relationship between intra (ICW) and extra (ECW) cellular fluids [11] as well as linked to cellular functions [12]. In this context, Pollastri et al. [13], showed that, during Giro d’Italia 2016, cyclists with better PhA performed higher mean maximal power (MMP) for 10 and 15 s. Moreover, BIVA changes in professional road cyclists over a short multistage race were correlated to the plasma volume changes [14]. Therefore, BIVA is already widely used in professional cyclists for performance assessment [13], training load monitoring [14] and body composition [15].

B-mode US analysis allows for the acquisition of high-resolution images of subcutaneous tissues, particularly adipose tissue [8]. In contrast, BIVA facilitates the classification and monitoring of changes in bioelectrical parameters, specifically those related to vector length and direction [10–11]. The combined use of these methods provides complementary data on body composition, enabling the assessment of both fat and lean soft tissue components. However, their application in sports science is largely limited to the use of US and BIA-derived data for estimating body composition variables, relying heavily on the predictive errors inherent in estimation Eqs. [7, 10]. Therefore, focusing these methodologies on the direct analysis of raw properties may represent a more precise approach for evaluating body composition.

In professional cycling, the pre-season phase is commonly an optimal period for body composition related interventions and training to optimize cyclists body shape. Despite that, to the best of our knowledge, no studies have monitored longitudinally changes in body composition and performance throughout the pre-season period in professional cyclists. On these bases, the aim of the present study was to monitor cyclists’ body composition using methods that allow for the assessment of raw body composition measurements, such as US-derived subcutaneous adipose tissue and BIVA patterns. We hypothesized that body composition changes could occur during the pre-season, leading to a reduction of adipose tissue, changes in soft tissues and fluids status according to changes in training load and performance parameters.

## Methods

### Participants

Twelve professional male cyclists participated in the study (mean ± SD: age 23 ± 4 years, body mass 65.1 ± 5.4 kg, stature 1.75 ± 0.05 m). Participants were classified as Tier 4 (Elite/Professional Level) according to the Participant Classification Framework (McKay et al., 2021). Before participating in the study, all participants provided written informed consent by signing a Consent Form. The study was approved by the Regional Health Unit Authority Ethics Committee (code: AGBMG23) and conducted in conformity with the Declaration of Helsinki.

### Study design

Body composition and field performance data were measured by the same operator on three occasions over approximately three months during the pre-season. The initial body composition assessment took place at the start of the pre-season training camp in December (T1). A second assessment was conducted 33 days later, during the second pre-season training camp in January (T2), and the final measurement was carried out 36 days after T2, during the last pre-season training camp in February (T3). Performance data for T1 were derived from an analysis of training data from the 30 days preceding the first measurement. For T2 and T3, training data were evaluated for the periods between T1 and T2, and T2 and T3, respectively.

### Anthropometric measurements

Anthropometric variables, including height, body mass, and body circumferences, were measured before breakfast. Body mass was recorded to the nearest 0.1 kg using a calibrated digital scale (GIMA, Gessate MI, Italy). Standing height was measured to the nearest 0.5 cm with a manual stadiometer (GIMA, Gessate MI, Italy). Body circumferences were assessed to the nearest 0.1 cm using a flexible tape measure (GIMA, Gessate MI, Italy).

### Ultrasound measurements

**Fig. 1:**
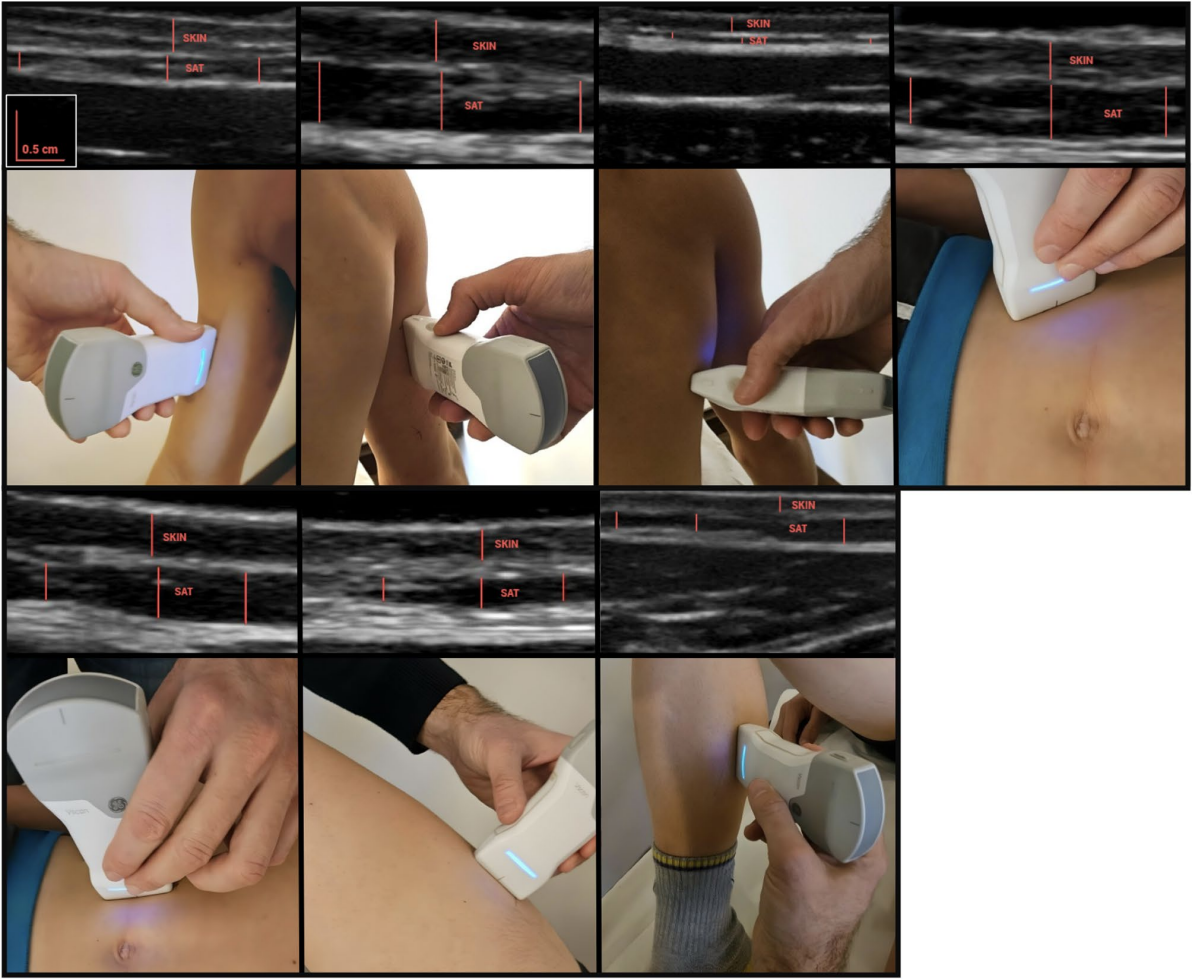
Sites of ultrasound measurements

As described in O’Neill et al. [6], SAT thickness was measured to the nearest 0.1 mm at seven ISAK (International Society for the Advancement of Kinanthropometry) recognized sites: triceps, biceps, subscapular, supraspinal, abdominal, anterior thigh, and medial calf. On each occasion, the same operator identified and marked these sites on the right-hand side of the body with a felt-tip pen. For consistency and to ensure ease of access during scanning, the biceps, triceps, and subscapular sites were imaged with the athlete seated, while the supraspinal, abdominal, anterior thigh, and medial calf sites were assessed with the athlete supine. Upper limb sites were measured with the elbow fully extended, while lower limb sites were measured with the knee flexed to 90 degrees. All ultrasound measurements were conducted longitudinally using the Vscan Air CL B-Mode ultrasound system (GE HealthCare, Chicago, IL, USA) with a linear array transducer (3–12 MHz) set to the general musculoskeletal setting. A 3–5 mm layer of ultrasound transmission gel (GIMA, Gessate MI, Italy) was applied, with the probe held parallel to the skinfold, centered over the inked site, and resting on the thick layer of gel to avoid compressing the underlying skin and subcutaneous adipose tissue, as shown in Fig. 1.

Three US images were taken at each measurement site, and the thickness of the SAT thicknesses, measured from the inferior border of the dermis to the fascial layer surrounding the adjacent muscle, was recorded to the nearest 0.1 mm. The average of the three measurements was used to determine the SAT thickness. All US images were saved and later analyzed using electronic calipers in ImageJ (version 1.46r, U.S. National Institutes of Health, Bethesda, Maryland, USA). Fat mass percentage (FM%) was calculated using the formula provided by O’Neill et al. [6].

### Bioimpedance measurements

Bioelectrical impedance was measured with a phase-sensitive impedance device (BIA 101 BIVA PRO, Akern System, Firenze, Italy) at 50 kHz frequency. Standard whole-body tetrapolar measurements were performed in compliance with the manufacturer’s guidelines and analysed according to the BIVA method [16]. BIA was performed in the morning, before breakfast. Before the measurement, participants rested in a supine position for one minute and emptied their bladder. All measurements were carried out in a temperature-controlled room.

### Training load and field-based performance parameters

Power meters (Favero Assioma Duo, Favero Electronics srl., 84 Arcade, TV, Italy) and GPS units (Bryton S800, Bryton 86 Inc., Taipei City, Taiwan) were used to collect data. All cyclists were informed about the importance of zero calibration for power meters before each ride. Duration, distance, elevation gain, and power data were manually analyzed using cycling software (WKO5; TrainingPeaks LLC, Boulder, CO). Erroneous outliers were identified and manually corrected using the Data Spike ID and FIX chart. If the number of outliers exceeded 5% of the total data to be analyzed, those values were eliminated, and the data were considered missing. Absolute and relative maximum mean power (MMP) values for 15 s, 5 min, and 20 min were calculated for all training sessions. Work and intensity were calculated as total work and the percentage of time (% time) spent in four power output bands: <1.9 W⋅kg⁻¹, 2.0–4.9 W⋅kg⁻¹, 5.0–7.9 W⋅kg⁻¹, and > 8.0 W⋅kg⁻¹ [17].

### Statistical analysis

All data were checked for normality using the Shappiro-Wilk test. Repeated Measures Analysis of Variance (ANOVA) tests were used to assess differences in body composition across time. Post-hoc analysis using pairwise comparison were performed using the Bonferroni correction. Partial eta squared (ηp²) effect sizes (ES) were reported to measure the magnitude of significant differences. Effect sizes greater than 0.01, 0.06, and 0.14 were considered small, moderate, and large, respectively [18]. A paired one-sample Hotelling’s T² test was used to identify bioelectrical changes over time, with statistical significance set at *p* < 0.05. Mahalanobis distance was calculated to assess ES, and threshold values were established for ES < 0.5 (small), ES ≥ 0.8 (medium), and ES ≥ 0.8 (large). Statistical analyses were performed using JASP 0.18.3 (JASP, Amsterdam, Netherlands). A significance level of *p* < 0.05 (two-tailed) was applied. Data are presented as mean ± standard deviation, unless otherwise stated.

## Results

### Ultrasound measurements

**Fig. 2:**
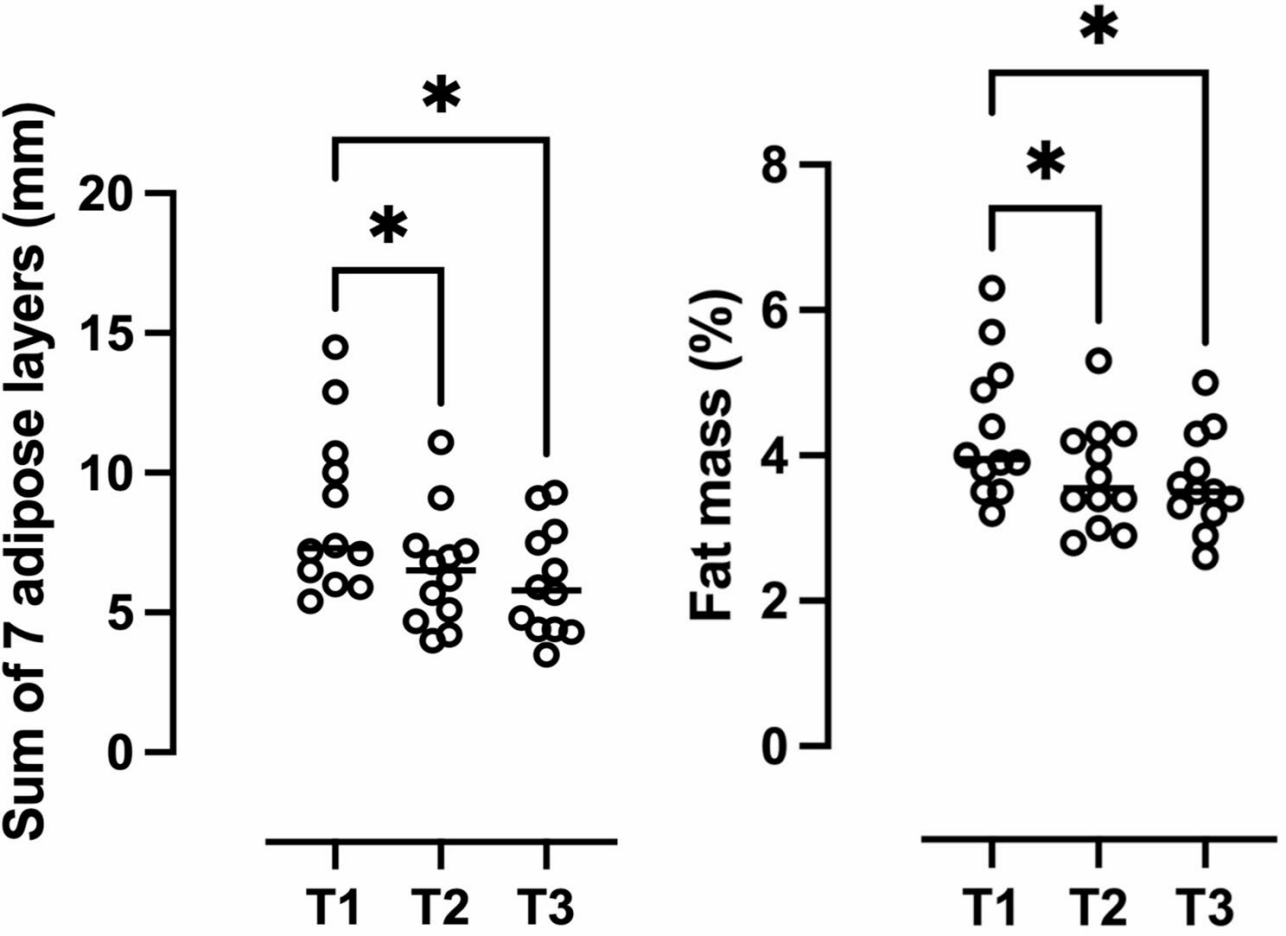
On the left site, the sum of 7 adipose thicknesses (∑ 7 US) during the pre-season. On the right side, the fat mass percentage during the pre-season. ^*^ denotes significant differences (*p* < 0.05) between timepoints

The sum of 7 adipose thickness (∑ 7 US) decreased across the pre-season (*p* = 0.001; ES = 0.45; Fig. 1): T3 (6.11 ± 1.96 mm) was lower than T2 (6.54 ± 2.07 mm, *p* = 0.010) and T1, (8.56 ± 2.93 mm *p* = 0.002), as shown in Fig. 2. FM% decreased across the pre-season (*p* = 0.005; ES = 0.38). T1 (4.35 ± 0.96%) was higher than T2 (3.72 ± 0.73%, *p* = 0.024) and T3 (3.63 ± 0.67%, *p* = 0.008) (Fig. 2). No differences were found between T2 and T3 (*p* = 1.000). Full disclosure of ultrasound variables can be found in Table 1.

**Table 1:** Body composition values assessed during the pre-season

	T1		T2		T3	
	Mean ± SD	Range	Mean ± SD	Range	Mean ± SD	Range
Body mass (kg)	65.4 ± 5.4	57.4–77.7	65.4 ± 5.3	59.5–77.8	64.6 ± 5.8	57.70–78.00
∑ seven ultrasound sites (mm)	8.56 ± 2.93	5.43–14.50	6.54 ± 2.07 ^α^	4.00-11.10	6.11 ± 1.96 ^α^	3.50–9.30
Fat mass (%)	4.35 ± 0.96	3.20–6.30	3.73 ± 0.73 ^α^	2.80- 5.30	3.63 ± 0.67 ^α^	2.60- 5.00
Resistance/h (Ω/h)	304.01 ± 27.92	251.2–340.00	305.62 ± 25.66	262.71–352.80	283.49 ± 25.55 ^α, β^	246.1-324.72
Reactance/h (Ω/h)	39.56 ± 4.11	38.39–3.22	38.39 ± 3.22	33.77–43.82	38.41 ± 3.63	32.43–44.41
Phase angle (°)	7.32 ± 0.29	6.80–7.80	7.12 ± 0.23	6.60–7.50	7.70 ± 0.39 ^α, β^	7.20–8.40
Impedance (Ω)	307.68 ± 26.35	264.8-343.15	308.22 ± 25.94	264.87-356.08	286.94 ± 26.04 ^α, β^	246.8-327.54

^α^ denotes significant differences from T1, ^β^ denotes significant differences from T2

**Fig. 3:**
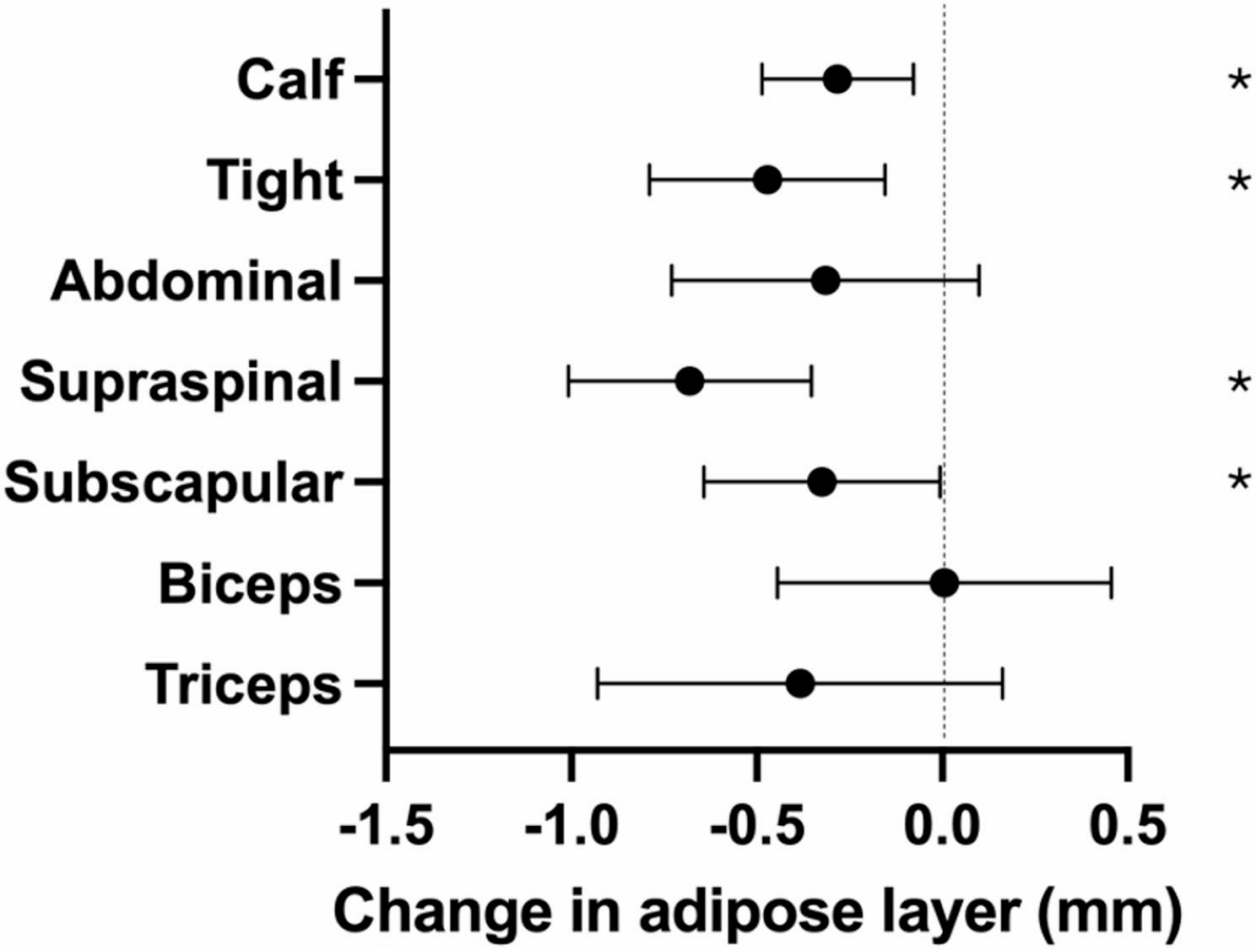
Change in the subcutaneous adipose thicknesses

Figure 3 shows the change in each site. The following adipose layers decreased during the pre-season significantly: calf (*p* = 0.001; ES = 0.46; thigh (*p* < 0.001; ES = 0.47); supraspinal (*p* < 0.001; ES = 0.58); and subscapular (*p* = 0.006; ES = 0.37). No significant changes were found in abdominal (*p* = 0.114); biceps (*p* = 0.672); and triceps (*p* = 0.226) adipose layers.

### Bioimpedance measurements

**Fig. 4:**
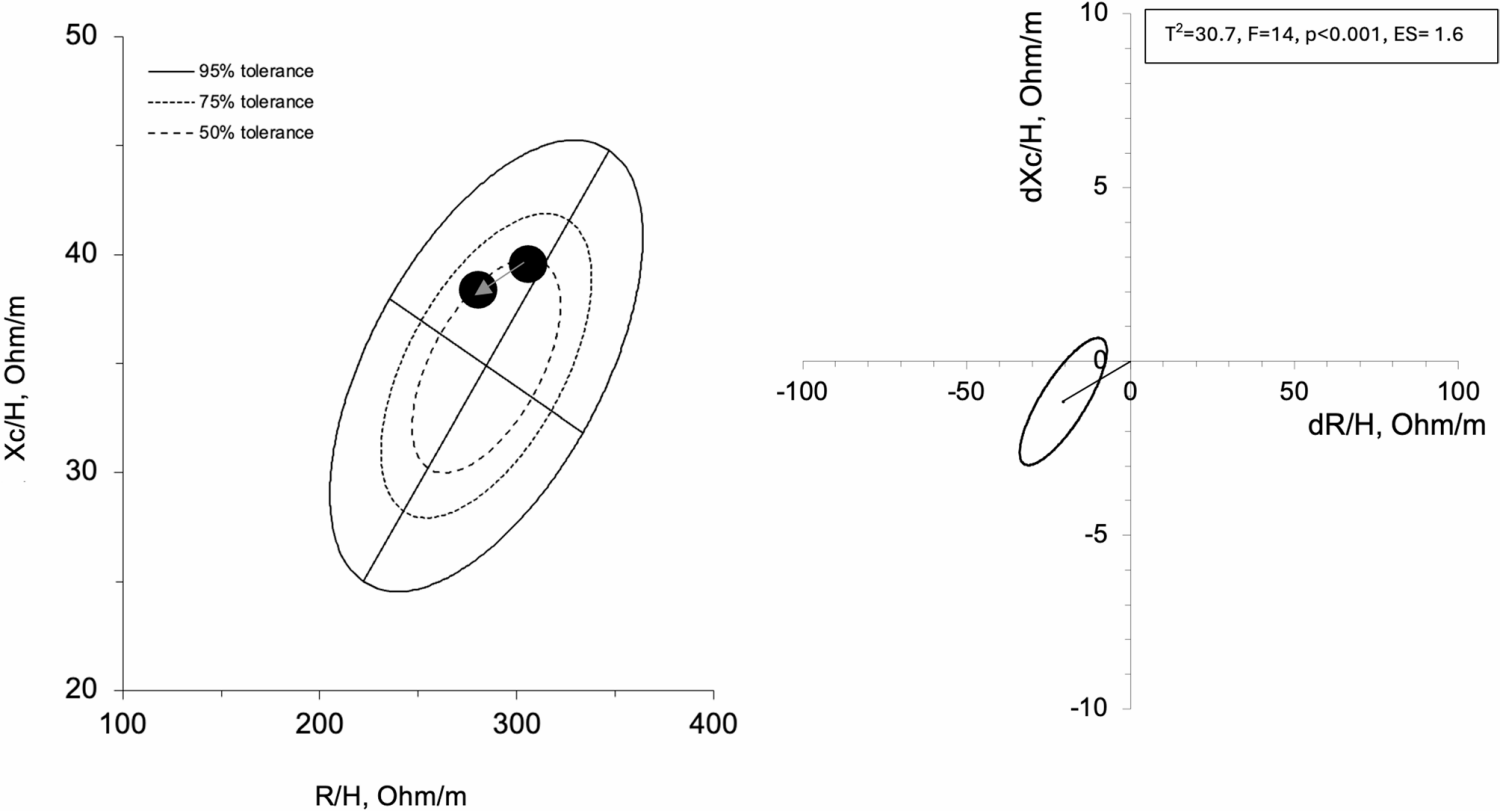
R-Xc, z-score, and paired graphs for the multivariate changes in bioelectrical parameters. In the left panels, bioimpedance data are plotted on the R-Xc z-score graph after the transformation of the impedance measurements from the athletes into bivariate z-scores. In the right panels, mean vector displacements with 95% confidence ellipses and results of the Hotelling’s T^2^ test are shown

PhA increased across the pre-season (*p* < 0.001; ES = 0.68). T3 (7.70 ± 0.39°) was higher than T2 (7.12 ± 0.23°, *p* = 0.001) and T1 (7.32 ± 0.30°, *p* = 0.001). Z decreases across the pre-season (*p* < 0.001; ES = 0.66): T3 (286.94 ± 26.04 Ω) was lower than T2 (308.22 ± *25*.*94* Ω, *p* = 0.001) and T1 (307.68 ± 26.35 Ω, *p* = 0.001), as shown in Table 1; Fig. 4.

### Training load parameters

The following parameters increased during the pre-season: duration (*p* < 0.001; ES = 0.79); distance (*p* < 0.001; ES = 0.89); absolute and relative work (*p* < 0.001; ES = 0.83; and *p* < 0.001; ES = 0.84, respectively) elevation gain (*p* < 0.001; ES = 0.82); % time spent between 2 and 4.9 W⋅kg^− 1^ (*p* = 0.028; ES = 0.28). % time spent between 5 and 7.9 W⋅kg^− 1^ (*p* < 0.001; ES = 0.61); % time spent above 8 W⋅kg^− 1^ (*p* < 0.001; ES = 0.63). No significant changes were found in time spent below 1.9 W⋅kg^− 1^ (*p* = 0.119). Full disclosure of performance variables can be found in Table 2.

**Table 2:** Training load parameters during the pre-season

	T1		T2		T3	
	Mean ± SD	Range	Mean ± SD	Range	Mean ± SD	Range
Distance (km)	1963 ± 507	635–2723	2245 ± 631	595–2830	3542 ± 224 ^α, β^	3045- 3852
Duration (min)	4092 ± 1081	1252–5493	4541 ± 1244	1222–5615	6579 ± 490 ^α, β^	5845- 7587
Elevation gain (m)	25,027 ± 6396	10,004–36,475	32,272 ± 10,293 ^α^	5817–42,223	43,619 ± 6731 ^α, β^	29,857–53,380
Absolute Work (kJ)	44,261 ± 11,953	13475-62,206	54,104 ± 16,270 ^α^	12,319–68,941	80,055 ± 7865 ^α, β^	64,167–89,380
Relative Work (kJ⋅kg^− 1^)	678 ± 196	235–1013	830 ± 262	204–1140	1245 ± 126 ^α, β^	1004- 1456
Time in zone 0–1.9 W⋅kg^− 1^ (%)	25.3 ± 8.2	11.6–35.4	22.8 ± 6.8	10.8–30.4	22.7 ± 6.1	12.1- 29.9
Time in zone 2–4.9 W⋅kg^− 1^ (%)	67.8 ± 9.2	59.5–86.1	69.1 ± 8.8	59.7–88.3	65.6 ± 7.8 ^β^	53.9- 82.5
Time in zone 5–7.9 W⋅kg^− 1^ (%)	6.40 ± 2.30	1.90–10.50	7.70 ± 3.64	0.7–13.0	10.6 ± 3.0 ^α, β^	4.8- 15,5
Time in zone > 8 W⋅kg^− 1^ (%)	0.5 ± 0.3	0.0- 1.0	0.4 ± 0.3	0.1- 0.7	1.1 ± 0.5 ^α, β^	0.1- 2.0

^α^ denotes significant differences from T1, ^β^ denotes significant differences from T2

### Field-based performance parameters

Neither significant changes in absolute nor relative MMP 15 s were found (*p* = 0.230 and *p* =, 0.078, respectively). The following parameters improved during the pre-season: absolute and relative MMP 5 min (*p* = 0.014; ES = 0.32, and *p* = 0.003; ES = 0.42, respectively); absolute and relative MMP 20 min (*p* < 0.001; ES = 0.80, and *p* < 0.001; ES = 0.78, respectively); absolute and relative MMP 60 min (*p* < 0.001; ES = 0.82, and *p* < 0.001; ES = 0.84, respectively). Full disclosure of performance variables can be found in Table 3.

**Table 3:** Field-based parameters during the pre-season

	T1		T2		T3	
	Mean ± SD	Range	Mean ± SD	Range	Mean ± SD	Range
MMP 15 s (W)	1008 ± 232	411–1391	977 ± 157	667–1190	943 ± 164	748- 1265
MMP 5 min (W)	392 ± 40	295- 473	398 ± 41	289- 461	413 ± 30 ^α^	366- 486
MMP 20 min (W)	308 ± 30	249- 380	322 ± 35	239- 383	361 ± 29 ^α, β^	326- 418
MMP 60 min (W)	249 ± 17	215- 276	265 ± 25	196–303	305 ± 28 ^α, β^	256- 351
Relative MMP 15 s (W⋅kg^− 1)^	15.3 ± 3.2	7.2- 19.7	15 ± 2.2	11- 17.5	14.6 ± 1.9	11.6- 17.5
Relative MMP 5 min (W⋅kg^− 1^)	5.98 ± 0.34	5.14–6.42	6.10 ± 0.53	4.79–6.96	6.41 ± 0.28 ^α, β^	6.02- 6.82
Relative MMP 20 min (W⋅kg^− 1^)	4.71 ± 0.22	4.34–4.97	4.93 ± 0.39	3.96–5.45	5.61 ± 0.45 ^α, β^	4.91- 6.36
Relative MMP 60 min (W⋅kg^− 1^)	3.81 ± 0.19	3.55–4.24	4.07 ± 0.32 ^α^	3.25–4.42	4.73 ± 0.36 ^α, β^	4.21- 5.19

^α^ denotes significant differences from T1, ^β^ denotes significant differences from T2

## Discussion

This study provides new insights into the relationship between body composition, training load, and performance parameters during the pre-season in professional road cyclists. The main finding is that cyclists experienced a reduction in subcutaneous adipose tissue thickness, along with gains in fluid and soft tissue, while training load and performance parameters increased throughout the pre-season period.

A significant reduction in the sum of 8 SAT layers was observed by the end of the pre-season (~ 28%), with the greatest decrease occurring at lower limb sites compared to trunk sites. Specifically, the reductions in thigh and calf sites were 31% and 27%, respectively, compared to the start of the pre-season. Endurance exercise induces whole-body fat mobilization, rather than fat loss from local adipose tissue depots adjacent to the working muscles. However, it is well known that during endurance exercise, both muscle temperature and energy demand increase, leading to a rise in blood flow to the muscles [19, 20]. This phenomenon may also affects the adjacent tissues, such as the SAT, by promoting the influx of lipolytic substances (e.g., catecholamines) to mobilize fat, providing energy to the adjacent contracting muscles [21]. Hence, it is possible that the increased blood perfusion during exercise in areas where it is most needed, specifically, the SAT of the lower limbs, created more favourable conditions for the release and transport of body fat, which could have contributed to muscle fat oxidation [19, 20]. Regarding the estimated FM, they may appear lower compared to other values reported in the literature [15]. This could be due to a tendency of the O’Neill Eq. [6] to underestimate body fat levels, particularly in individuals with low body fat, such as cyclists [15]. These factors might lead practitioners to prefer monitoring raw measures (the sum of SAT layers at specific sites) rather than relying on predictive formulas, thus avoiding potential estimation errors.

Along with the decrease of SAT seen with US, the BIVA of our cyclists showed changes in terms of body water content. The Rx-Xc graph indicates a progressive significant decrease of the mean vector from the top to the bottom along the major axis together with the shift to the left along the minor axis at the end of the pre-season. These changes suggest that the cyclists increased their extracellular fluids, probably due to plasma volume expansion promoted by the increase of training load as showed during stages races [13, 14] and in other sports [22, 23]. Together with the extracellular fluid changes, the vector shift to the left along the minor axis of the Rx-Xc graph along with the PhA increase indicates an increase of the cellular function and then muscle hypertrophy [13, 24].

Pre-season training aims to develop the physical requirements needed to face the competitive season workload, therefore distance, elevation gain, and work increased progressively up to the competitive season [2, 25]. In this scenario, long aerobic-based training sessions were performed during the pre-season, with a progressive increase of time spent at 2–4.9 W⋅kg^− 1^ from T2 to T3. Cyclists rode around 22–25% of their training time at intensities below under 2 W⋅kg^− 1^ without significant changes, probably due to non-pedalling activity and the nature of stochastic power output [26, 27]. Beside the aerobic-based training, riders’ time spent at high (5–7.9 W⋅kg^− 1^) and very high intensities (> 8 W⋅kg^− 1^) increased, approaching the competitive season. This progressive increase on high intensities training load is fundamental for performance progression and propaedeutic to face race pace and decisive attacks during races [26, 28, 29]. Consequently, cyclists’ power profile increased for the 5-min, 20-min, and 60-min mean maximal power outputs, both in absolute and relative terms. However, 15-s performance did not increase. Therefore, threshold and endurance were improved but not sprint. The improvement in such high-power output requires demanding sprint- and interval training sessions, in cycling these intensive sessions are mostly performed during competitions, because it has been reported to be less psychologically demanding, and the presence of competitors enhances motivation and reduces perception of effort [26, 29]. Therefore, the lack of improvement in high power output values during the pre-season could be justified by that.

During the pre-season, professional cyclists participate in training camps that provide a more comprehensive support network than what is typically available at home. These camps offer access to a range of specialists, including mechanics, nutritionists, sports therapists, chefs, physicians, and coaches. This structured and resource-rich environment is meticulously designed to target specific weaknesses, facilitating individualized performance improvements [2, 3]. Within this “protected setting,” riders are generally more inclined to tolerate higher training loads, supported by personalized nutritional strategies. The structured routine of training sessions and meals, coupled with constant access to team staff, fosters greater adherence to both training and dietary protocols.

As outlined above, pre-season training for cyclists predominantly involves prolonged aerobic-based sessions conducted at intensities ranging from 2.0 to 4.9 W·kg⁻¹. These sessions predominantly utilize fat oxidation as the primary energy substrate. Alongside endurance workouts, athletes also engage in high-intensity intervals at intensities exceeding 8.0 W·kg⁻¹, where energy demands are predominantly met through anaerobic and aerobic glycolysis. Additionally, the reduction in body fat is influenced by various factors; however, it is well-established that both the volume and intensity of training, combined with a sustained energy deficit, are pivotal [3]. In our cyclists, the observed decrease in body fat is most likely attributable to the combination of prolonged aerobic training and a carefully managed caloric intake designed to achieve a negative energy balance.

We acknowledge the observational nature of the study, which represents a limitation. Additionally, analyzing the relationship between training load parameters and body composition using a larger sample size might have provided more comprehensive insights into this topic. Similarly, evaluating energy intake could have offered further information on changes in body composition. Nonetheless, the sample consisted of professional cyclists, and all evaluations were conducted using standardized procedures. Despite the inherent limitations of analyzing field-based power output data, we implemented strict methodological protocols, such as calibrating power meters and applying data filtering, to ensure the collection of high-quality data. Future studies should include larger sample size and the analysis of energy balance. Lastly, hydration status was not directly assessed. However, the measurements were conducted away from exercise and after a night of rest following a balanced dinner. This context should be sufficient to ensure the absence of any significant alterations in the hydration of the soft tissues of the participants [16]. Controlled laboratory settings are necessary to further clarify the relationship between changes in body composition and training load parameters, as numerous influencing factors during real-world training, such as nutrition, environmental conditions, and slipstreaming, are difficult to assess comprehensively.

### Practical applications

For the first time it has been described the body composition changes along with training load and performance parameters during the pre-season in professional cyclists. Moreover, this investigation showed that US and BI are valuable methods to identify body composition changes, mainly when sophisticated methods are not available. These tools allow qualitative and semiquantitative assessments of adipose tissue and body fluids and soft tissue in endurance athletes, providing practical insights for the staff in terms of body composition assessment, and training load.

## Conclusions

Professional road cyclists showed a decrease of the adiposity, mainly in the lower limb together with the increase of soft tissue and extracellular fluids during the pre-season. The combination of US and BI through BIVA allows a valuable method for the longitudinal monitoring of body composition. Furthermore, training load increased, and performance parameters were improved. These findings may help coaches, sports scientists, and medical staff to evaluate body composition and its changes according to the training load in the field.

## Data Availability

Data are available from the corresponding author on request.
